# Accurate photosynthetic parameter estimation at low stomatal conductance: effects of cuticular conductance and instrumental noise

**DOI:** 10.1007/s11120-024-01092-8

**Published:** 2024-05-03

**Authors:** Syed Bilal Hussain, Joseph Stinziano, Myrtho O. Pierre, Christopher Vincent

**Affiliations:** 1 Citrus Research and Education Center, University of Florida, Lake Alfred, FL 33850 USA; 2 School of Agriculture and Food Science, University College Dublin, Dublin 4, Ireland; 3 Department of Biology, University of New Mexico, Albuquerque, NM USA; 4 Present Address: Plant Health Science Directorate, Canadian Food Inspection Agency, 1400 Merivale Rd, Ottawa, ON K2C 4B5 Canada

**Keywords:** Gas exchange, Stomatal conductance, Cuticular conductance, RACiR *A/C*_i_, Steady-state *A/C*_i_

## Abstract

Accurate estimation of photosynthetic parameters is essential for understanding plant physiological limitations and responses to environmental factors from the leaf to the global scale. Gas exchange is a useful tool to measure responses of net CO_2_ assimilation (*A*) to internal CO_2_ concentration (*C*_i_), a necessary step in estimating photosynthetic parameters including the maximum rate of carboxylation (*V*_cmax_) and the electron transport rate (*J*_max_). However, species and environmental conditions of low stomatal conductance (*g*_sw_) reduce the signal-to-noise ratio of gas exchange, challenging estimations of *C*_i_. Previous works showed that not considering cuticular conductance to water (*g*_cw_) can lead to significant errors in estimating *C*_i_, because it has a different effect on total conductance to CO_2_ (*g*_tc_) than does *g*_sw_. Here we present a systematic assessment of the need for incorporating *g*_cw_ into *C*_i_ estimates. In this study we modeled the effect of *g*_cw_ and of instrumental noise and quantified these effects on photosynthetic parameters in the cases of four species with varying *g*_sw_ and *g*_cw_, measured using steady-state and constant ramping techniques, like the rapid *A*/*C*_i_ response method. We show that not accounting for *g*_cw_ quantitatively influences *C*_i_ and the resulting *V*_cmax_ and *J*_max_, particularly when *g*_cw_ exceeds 7% of the total conductance to water. The influence of *g*_cw_ was not limited to low *g*_sw_ species, highlighting the importance of species-specific knowledge before assessing *A*/*C*_i_ curves. Furthermore, at low *g*_sw_ instrumental noise can affect *C*_i_ estimation, but the effect of instrumental noise can be minimized using constant-ramping rather than steady-state techniques. By incorporating these considerations, more precise measurements and interpretations of photosynthetic parameters can be obtained in a broader range of species and environmental conditions.

**Supplementary Information:**

The online version contains supplementary material available at 10.1007/s11120-024-01092-8.

## Introduction

Gas exchange is a powerful tool in plant physiology that allows us to understand CO_2_ and water relations in plants to compare plant performance in a range of environments (Stinziano and Way 2017; Vincent et al. 2017; Smith et al. 2020), to describe how plants cope with challenging environments (Kumarathunge et al. 2019; Zhu et al. 2020), and to model plant-environment interactions up to the global scale (Oleson et al. 2013; Rogers et al. 2017; Lombardozzi et al. 2018). However, gas exchange measurements require a sufficient signal-to-noise ratio to obtain high-quality data for inferences. This can be achieved by ensuring stomata are adequately open or by incorporating a model that accounts for cuticular conductance (*g*_cw_) (Márquez et al. 2021). However, many lines of research specifically address either species or conditions that produce low stomatal conductance, including xerophytic species, conditions of water deficit, or high temperatures. A comprehensive understanding of the factors affecting gas exchange measurements, including stomatal conductance (*g*_sw_) and *g*_cw_, is crucial for obtaining accurate data and drawing sound conclusions (See Table 1 for a list of abbreviations and equations).

**Table 1:** Abbreviations, variables, and equations used to calculate conductances and estimate net assimilation and internal CO_2_

Abbrev	Variable	Standard equation	Corrected equation
*A*	Net assimilation of CO_2_		
*C*_a_	Atmospheric concentration of CO_2_		
*E*	Transpiration		
*g*_lw_	Leaf conductance to water	${g}_{lw}\approx {g}_{sw}$	${g}_{lw}={g}_{sw}+{g}_{cw}$
*g*_bw_	Boundary layer conductance to water		
*g*_sw_	Stomatal conductance to water	${g}_{sw}\approx {g}_{lw}$	${g}_{sw}={g}_{lw}-{g}_{cw}$(3)
*g*_cw_	Cuticular conductance to water	Calculated according to Marquez et al. (2021)	
*g*_tc_	Total (leaf and boundary layer) conductance to CO_2_	${g}_{tc}=\frac{1}{\left(K+1\right)\frac{1.6}{{g}_{sw}}+\frac{1.37}{{g}_{bw}}}+\frac{K}{\left(K+1\right)\frac{1.6}{{g}_{sw}}+K\frac{1.37}{{g}_{bw}}}$(2)	
*g*_lc_	Leaf conductance to CO_2_	${g}_{lc}\approx \frac{{g}_{sw}}{1.6}$	${g}_{lc}=\frac{{g}_{sw}}{1.6}+\frac{{g}_{cw}}{20}$(4)
*g*_sc_	Stomatal conductance to CO_2_	${g}_{sc}=\frac{{g}_{sw}}{1.6}$	
*g*_cc_	Cuticular conductance to CO_2_	${g}_{cc}=\frac{{g}_{cw}}{20}$	
*C*_i_	Leaf internal CO_2_	${C}_{i}=\frac{\left({g}_{tc}-\frac{E}{2}\right){C}_{a}-A}{\left({g}_{tc}+\frac{E}{2}\right)}$(1)	
*C*_i uncor_	Leaf internal CO_2_ based on standard *g*_tc_		
*C*_i cor_	Leaf internal CO_2_ based on corrected *g*_tc_		
*C*_i mis_	Proportional misestimation of *C*_i_ due to ignoring *g*_cw_	${C}_{i mis}=\frac{{C}_{i uncorr}{-C}_{i cor}}{{C}_{i cor}}$ (5)	

Numbers in parentheses represent equation numbers in the manuscript

Stomatal conductance is a vital process that has a direct impact on plant growth and productivity, as it constrains photosynthesis and water-use. Plant species present a wide variation in *g*_sw_ depending on leaf morphology, physiology, ecotype, and environment, with many exhibiting low conductance. Low *g*_sw_ presents a challenge to the estimation of photosynthesis, particularly in C3 species with maxima below 0.2 mol m^−2^ s^−1^ (Körner et al. 1986; Tezara et al. 1998), and rapid stomatal closure whenever conditions are not ideal (Radin et al. 1994) or at a particular time of day (Steppe et al. 2006). For example, in a survey of *Citrus* x *sinensis* (L.) and *C. reticulata* (Blanco), 35% of the mid-morning (9:00 am-11:00 am) gas exchange measurements exhibited total leaf conductance to water (*g*_lw_) below 0.08 mol m^−2^ s^−1^ (Vincent et al., *unpublished data*). When *g*_lw_ is low, the signal-to-noise ratio in gas exchange measurements is also low. Additionally, it becomes difficult to obtain a sufficient range in intercellular CO_2_ concentration (*C*_i_) to characterize the response of net CO_2_ assimilation (*A*) to *C*_i_ (*A*/*C*_i_ curves) (Stinziano et al. 2020). Further compounding the signal-to-noise challenge in the context of *A*/*C*_i_ curves, plants respond to elevated CO_2_ by stomatal closure on an order of minutes.

Using *A*/*C*_i_ curves, we can apply the Farquhar-von Caemmerer-Berry model of photosynthesis (Farquhar et al. 1980) to estimate photosynthetic parameters for comparing plant performance (Way and Sage 2008), understanding photosynthetic acclimation (Kumarathunge et al. 2019), and modelling vegetative carbon uptake (Oleson et al. 2013). In species or conditions with low *g*_*sw*_ or stomata that close rapidly at high CO_2_, such as those seen in evergreen broadleaved species, these responses are difficult to characterize (Lin et al. 2023). Stomatal limitations may further lead to incorrect conclusions on what is limiting photosynthesis. For example, it is possible to misinterpret a stomatal limitation as a constraint related to Rubisco carboxylation. The recent development of rapid *A*/*C*_i_ response (RACiR) curves (Stinziano et al. 2017, 2019a, b) and subsequently of the dynamic acclimation approach which allows more efficient use of constant-ramping methods (Saathoff and Welles 2021) may be sufficiently fast to circumvent the difficulties of stomatal closure in such species.

Although *g*_lw_ is often assumed equal to *g*_sw_, this is not entirely accurate. Cuticular conductance (*g*_cw_) is a component of *g*_lw_ and a key factor that impacts gas exchange measurements in plants, as it involves the diffusion of gases across the cuticle layer of leaves (Márquez et al. 2022). Although in practice it is usually not considered, *g*_cw_ can impact *A*/*C*_i_ measurement, because the ratio of conductance to H_2_O and to CO_2_ is dramatically different between stomata and cuticles. The diffusivity of H_2_O and CO_2_ through the stomata have a ratio of 1.6, which forms the basis of estimates of stomatal conductance to CO_2_. However, the cuticle is a stronger barrier to CO_2_ than H_2_O where the diffusivity of H_2_O is much higher than that of CO_2_ with a ratio of 1:20–40 (Boyer et al. 1997; Boyer 2015b). In addition, overlooking *g*_cw_ can be problematic because, in certain cases, it can reach levels as high as 28% of *g*_lw_ (Holmgren et al. 1965; Boyer et al. 1997). Because estimates of *C*_i_ are based on total conductance to CO_2_ (*g*_tc_), assuming that *g*_lw_ = *g*_sw_ can lead to misestimating *C*_i_ (Table 1; Boyer 2015b). Variations in *g*_cw_ among plant species can significantly affect the accuracy of gas exchange measurements (Márquez et al. 2021; Boyer et al. 1997), particularly in low-conductance species (Márquez et al. 2022).

Previous studies have highlighted the impact of *g*_cw_ on gas exchange measurements and underscored the need for appropriate correction methods to ensure accurate measurements (Grassi and Magnani 2005; Tominaga et al. 2018). However, estimating *g*_cw_ separately from *g*_sw_, which usually governs gas diffusion through the leaf surface under illuminated conditions, has been a major challenge. Consequently, it is often neglected in gas exchange calculations. In this regard, a recent study by Márquez et al. (2022) has proposed a new approach, called the red-light method, which estimates *g*_cw_ from gas exchange measurements and a known CO_2_ concentration within the leaf during photosynthetic induction under red light. This novel method enables accurate estimation of *g*_cw_, the inclusion of which can improve the precision of gas exchange measurements. Lamour et al. (2022) recently provided a theoretical reassessment of the impact of *g*_cw_ in the measurement of *A*/*C*_i_ curves and the parameters calculated from them, using real *A*/*C*_i_ curves and applying a range of hypothetical *g*_cw_. In practice, many researchers face species or conditions of highly variable *g*_lw_. Thus, knowledge of the impact of these factors and how to circumvent the resulting limitations in practice is essential, especially under low *g*_lw_ conditions.

In this work, our objective was to assess the impact of *g*_cw_, *g*_sw_, and other sources of error on the validity of estimates of photosynthetic parameters. To test the impacts of *g*_cw_ and *g*_sw_ we selected four species that vary in both. We used the red-light method of *g*_cw_ measurement and steady state and RACiR methods to estimate *A*/*C*_i_-based parameters in these species with varying *g*_cw_ and *g*_sw_. Although the RACiR method has been superseded by a dynamic assimilation technique (DAT; Tejera-Nieves et al. 2024), which eliminated the need for running empty-chamber curves, the utility of both RACiR and DAT in terms of the use of constant-ramping and frequent logging to assess *A*/*C*_i_ are interchangeable. We modeled the impact of *g*_lw_ and *g*_cw_ on *C*_i_ estimates and developed criteria to understand the impact of conductance on the reliability of *A*/*C*_i_ curves and the resulting parameters and provide this model for future work.

## Materials and methods

### ***Modeling the impact of stomatal and cuticular conductance on estimates of internal CO***_***2***_

To estimate the impact of *g*_lw_ on the estimation of *C*_i_ we modeled the estimation of *C*_i_ in response to varying external CO_2_ concentrations (*C*_a_) and *g*_sw_. To achieve this we used the Photosyn() command in the {plantecophys} package (Duursma 2015). This model allowed us to fix vapor pressure deficit (1.5 kPa) and *C*_a_ (100, 400, or 2000 ppm), along with temperature (25 °C) and maximum rates of carboxylation (50 µmol m^−2^ s^−1^) and electron transport (100 µmol m^−2^ s^−1^), which are the default values in the model. We considered a range of *g*_lw_ values from 0.001 to 1 mol m^−2^ s^−1^ in increments of 0.001 mol m^−2^ s^−1^ to cover the full range of expected values from medium to low conductance species. *C*_i_ was initially calculated according to the standard calculations of most gas exchange equipment. This calculation is based on von Caemmerer and Farquhar (1981) as:

1 $${C}_{i}=\frac{\left({g}_{tc}-\frac{E}{2}\right){C}_{a}-A}{\left({g}_{tc}+\frac{E}{2}\right)}$$

where *g*_tc_ is total conductance to CO_2_, which was estimated as:

2 $${g}_{tc}=\frac{1}{\left(K+1\right)\frac{1.6}{{g}_{sw}}+\frac{1.37}{{g}_{bw}}}+\frac{K}{\left(K+1\right)\frac{1.6}{{g}_{sw}}+K\frac{1.37}{{g}_{bw}}}$$

where 1.6 and 1.37 are the ratios of diffusivity of CO_2_ to H_2_O in air (in stomata) and in the boundary layer. *K* is the ratio of stomatal resistance of the adaxial to abaxial sides of the leaf. For this consideration, we set *K* = 0, which is common for low conductance species, and boundary layer conductance to water (*g*_bw_) as 2.23 mol m^−2^ s^−1^. These equations are standard for estimates in gas exchange. If *K* = 0 and *g*_bw_ is large enough to be ignored, as is expected in most gas exchange measurement contexts (Márquez et al. 2021), then Eq. 2 can be simplified as: ${g}_{tc}=\frac{{g}_{sw}}{1.6}$, which assumes that *g*_lw_≈*g*_sw_.

At high *g*_lw_, this assumption may be roughly correct. However, as *g*_lw_ decreases relative to *g*_cw_, the assumption is likely to become less tenable and increasingly impact estimates of *C*_i_, because the cuticular conductance to CO_2_ (*g*_cc_) is expected to be approximately 0.05 *g*_cw_ (Márquez et al. 2022). Thus, we corrected the original *g*_lc_ by calculating:

3 $${g}_{sw}={g}_{lw}-{g}_{cw}$$

4 $${g}_{lc}=\frac{{g}_{sw}}{1.6}+\frac{{g}_{cw}}{20}$$

where 1:20 is the expected proportion of *g*_cw_ to *g*_cc_ (Boyer et al. 1997). Because the Photosynth() command assumes *g*_lw_ = *g*_sw_ and does not allow explicit parameterization of *g*_tc_, but calculates *g*_tc_ = *g*_lw_/1.6 (Duursma 2015), we recalculated a pseudo-*g*_lw_ to result in the corrected *g*_tc_ by multiplying the corrected *g*_tc_ by 1.6. *C*_i_ was then recalculated based on the new estimate of *g*_lc_. We then calculated the proportional misestimation of *C*_i_ (*C*_i mis_) when not accounting for *g*_cw_ as the difference between uncorrected *C*_i_ (*C*_i uncorr_) and corrected *C*_i_ (*C*_i cor_) as a proportion of *C*_i_* C*_i cor_:

5 $${C}_{i mis}=\frac{{C}_{i uncorr}{-C}_{i cor}}{{C}_{i cor}}$$

### Plant material

Papaya (*Carica papaya* L.), bell pepper (*Capsicum annuum* L.), citrus (*C. x sinensis* cv. ‘Valencia’) and magnolia (*Magnolia grandiflora* L.) plants were grown in a greenhouse in Lake Alfred, FL 33850, USA (28.1021° N, 81.7121° W) in a natural diurnal cycle and daily maximum and minimum of ~ 25–34 °C. All plants were irrigated twice a week to ensure that the growing media 'Pro-mix BX' (Premier Tech Ltd, Quebec, Canada) remained adequately moist and were fertilized with 12-4-8 Miracle-Gro liquid fertilizer (The Scotts Company, LLC) every 2 weeks. SS and RACiR curves were performed on healthy and fully expanded leaves. In total, five plants each for bell pepper, magnolia, and papaya were measured, while 18 citrus plants were used.

### Gas exchange measurements

Response curves were measured using a portable infrared gas analyzer (LI-6800, Li-COR Inc., Lincoln, NE, USA) (Console version: Bluestem v.1.4.02) equipped with a 3 × 3 cm leaf chamber (9 cm^2^). Leaf chamber settings were as follows: fan speed of 10,000 rpm, flow rate of 300 μmol s^−1^ and 600 μmol s^−1^ (for RACiR and SS, respectively), overpressure of 0.1 kPa, 24 mmol mol^−1^ of H_2_O in the reference cell producing between ~ 63% and 79% relative humidity (RH) in the leaf chamber, and producing a variation of ~ 2 to 3% in RH over the course of measurements, photosynthetically active radiation of 1200 µmol m^−2^ s^−1^ [90% red and 10% blue light], and chamber air temperature at 25 °C. We first measured paired standard RACiR and SS to compare parameter estimates from each. Each set of RACiR and SS curves was gathered on adjacent leaves of the same plant (not on the same leaf to avoid the effects of the first curve on the results of the second) on the same day.

### ***Rapid A/C***_***i***_*** response (RACiR) curves***

RACiR data were gathered beginning at a reference CO_2_ concentration of 100 µmol mol^−1^ with a ramping rate of 100 µmol mol^−1^ min^−1^, ending at 900 µmol mol^−1^. After testing paired measurements, we gathered five additional RACiR curves from five plants each for all four species, this time the *C*_a_ range was 100 µmol mol^−1^ to 2000 µmol mol^−1^ with the same 100 µmol mol^−1^ min^−1^ ramping rate. Before each curve, the IRGA was matched to allow the system to stabilize in match mode. The empty chamber curves were determined in exactly the same way as the RACiR curves, except that the leaves were not clamped. Moreover, one empty chamber curve was used to calibrate the two leaf runs taken within 1 h. For all measurements, the leaf chamber was clamped on the leaf and allowed to acclimate to set environmental conditions until *A* reached a steady state before running the CO_2_ response curves; however, the acclimation time was variable among species. The ‘Autolog’ program of LI-6800 was used to record data every 2 s (9 min and 19 min for RACiR when CO_2_ concentration was set at 100–900 µmol mol^−1^ and 100–2000 µmol mol^−1^, respectively).

## ***Steady-state A***/***C***_i_***curves***

The LI-6800 CO_2_ response program was used for the steady-state (SS) *A*/*C*_i_ curves. Firstly, all environmental conditions were set the same as the RACiR curves except for the CO_2_. Secondly, the sample CO_2_ was set to 380 µmol mol^−1^ CO_2_. All leaves were acclimated to set environmental conditions until *A* reached a steady state before running the CO_2_ response curves. SS curves were collected starting at a reference CO_2_ concentration of 380 µmol mol^−1^, then proceeding through the following progression conditions: 285, 190, 145, 100, 50, 380, 475, 570, 665, 760, 960, 1160, 1460, 1760, 1960 µmol mol^−1^ CO_2_ with minimum and maximum waiting times of 60 s and 120 s, respectively. The reference and sample IRGAs were matched before each measurement (Stinziano et al. 2017), and the measuring time for each SS *A*/*C*_i_ curve was approximately 30 min.

### ***Measurement of cuticular conductance (g***_***cw***_***)***

The *g*_cw_ was estimated using the red-light technique introduced by Márquez et al. (2021). Briefly, the plants were dark-adapted for 12 h. A leaf was then placed in the darkened LI-6800 chamber and allowed to acclimate to 400 µmol mol^−1^ CO_2_, 25 °C, and 24 mmol mol^−1^ of H_2_O in the reference before recording dark respiration (*R*_dark_). The dark-acclimated leaf was subsequently exposed to the same environmental conditions as those for *R*_dark_ measurements, except that 100 µmol m^−2^ s^−1^ red light was applied, and measurements were taken every 7 s during the light induction until the maximum *A* was achieved, and over 5 min at steady state.

To determine the rate of electron transport or the electron transport rate at a given CO_2_ concentration (*J*_a_) and gamma star (Γ*, represents the CO_2_ photo-compensation point) during the experiment, a RACiR curve was performed under the same conditions as during the red-light induction, with the exception that stomatal opening is promoted using 100 µmol m^−2^ s^−1^ red-blue (40 µmol m^−2^ s^−1^ blue), and a CO_2_ concentration ranging from 100 µmol mol^−1^ to 1400 µmol mol^−1^ with a 100 µmol mol^−1^ min^−1^ ramping rate. The leaf chamber was clamped onto the leaf and allowed to acclimate to the set environmental conditions until *A* reached a steady state before running the CO_2_ response curves.

### Analysis

Raw data obtained for each leaf sample were filtered and corrected using the empty chamber data as per the protocol for RACiR curve correction provided by Coursolle et al. (2019). RACiR data were calibrated using the {racir} R package (Stinziano et al. 2019a; Lawrence et al. 2019; Stinziano 2020). All curves were fit using the fitaci() command from the {plantecophys} package (Duursma 2015; Pilon et al. 2018) in R statistical software (R Core Team 2013), with options selected to fit triose phosphate utilization (TPU) limitation rate and with no temperature correction. We extracted the maximum Rubisco carboxylation capacity (*V*_cmax_), maximum electron transport rate under saturating light (*J*_1200_), triose phosphate utilization (TPU), dark respiration (*R*_dark_), the *C*_i_ at the CO_2_ to RuBP transition (*C*_itrans1_), and the *C*_i_ at RuBP to TPU transition (*C*_itrans2_) for analysis. Subsequently, all *A*/*C*_i_ curves were fit with uncorrected and corrected *g*_sw_ values to assess the impact of *g*_cw_ on parameter estimates.

### Statistics

The resulting parameters from the fit curves were subjected to analysis of variance and paired Student’s *t*-tests by using Statistix 8.1® software to test for differences between SS and RACiR curves. These tests were performed on *A*/*C*_i_ curves derived with uncorrected and corrected *g*_sw_. Multiple comparisons were performed with Tukey’s honestly significant difference (Tukey’s HSD), and differences were significant when *P* < 0.05. Data in the figures were processed and analyzed with R (R version 4.1.1, 2021 "Kick Things").

### Data and code

Data and code are available in supplementary files S1.

## Results

### ***At low g***_***sw***_***, C***_***i***_*** estimates become unreasonable***

**Fig. 1:**
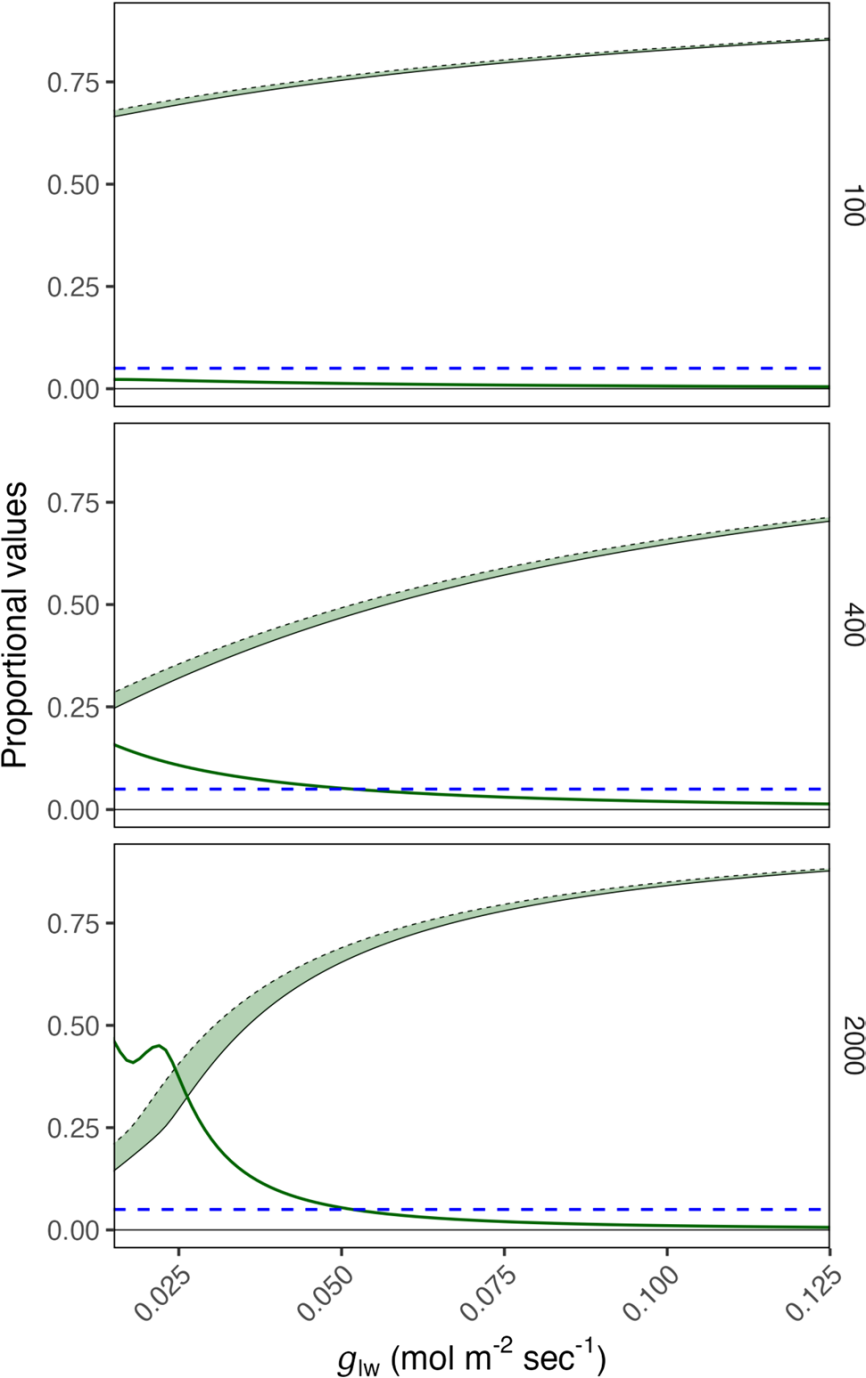
*C*_i_ in response to *g*_lw_, where *g*_lw_ is assumed to equal *g*_sw_, under varying *g*_lw_ and scenarios of *C*_a_ = 100, 400, or 2000 µmol mol^−1^. Proportional *C*_i_ values are proportional to the value when *g*_sw_ = 1 mol m^−2^ s^−1^. The light green ribbon shows the gap between the corrected and uncorrected *C*_i_ values, bounded on the bottom by the uncorrected *C*_i_ estimate (solid black line) and on the top by the corrected estimate (dashed black line). The correction accounts for the differential effect of *g*_cw_ in the estimation of *g*_cc_ and thus on *C*_i_. The solid green line shows the proportional misestimation of *C*_i_ due to not accounting for *g*_cw_ proportional to the corrected value at the same* g*_sw_. The dashed blue horizontal line denotes a proportion 0.05 (5% miscalculation or uncertainty). In all cases vapor pressure deficit was 1.5 kPa, temperature was 25 °C, maximum rate of carboxylation was 50 µmol m^−2^ s^−1^, electron transport was 100 µmol m^−2^ s^−1^, and *g*_cw_ was 0.0056 mol m^−2^ s.^−1^ (results for papaya, which fell toward the middle of the distribution among the four species)

There was a strong impact of *g*_lw_ on *C*_i_. *C*_a_ also impacted the response dynamics, increasing the magnitude of *C*_i_ reduction in response to reduced *g*_lw_ (Fig. 1). The impact of *g*_lw_ at a *C*_a_ of 100 ppm was negligible, but its impact was strong at both 400 and 2000 ppm. This result indicated limits to the estimation of *C*_i_ at low *g*_lw_, suggesting improvements in the accuracy of conductance estimates and *C*_i_ impact estimates of *C*_i_ to a greater degree at high *C*_a_.

### ***Cuticular conductance impacts estimation of internal CO***_***2***_*** as a proportion of total leaf conductance***

**Fig. 2:**
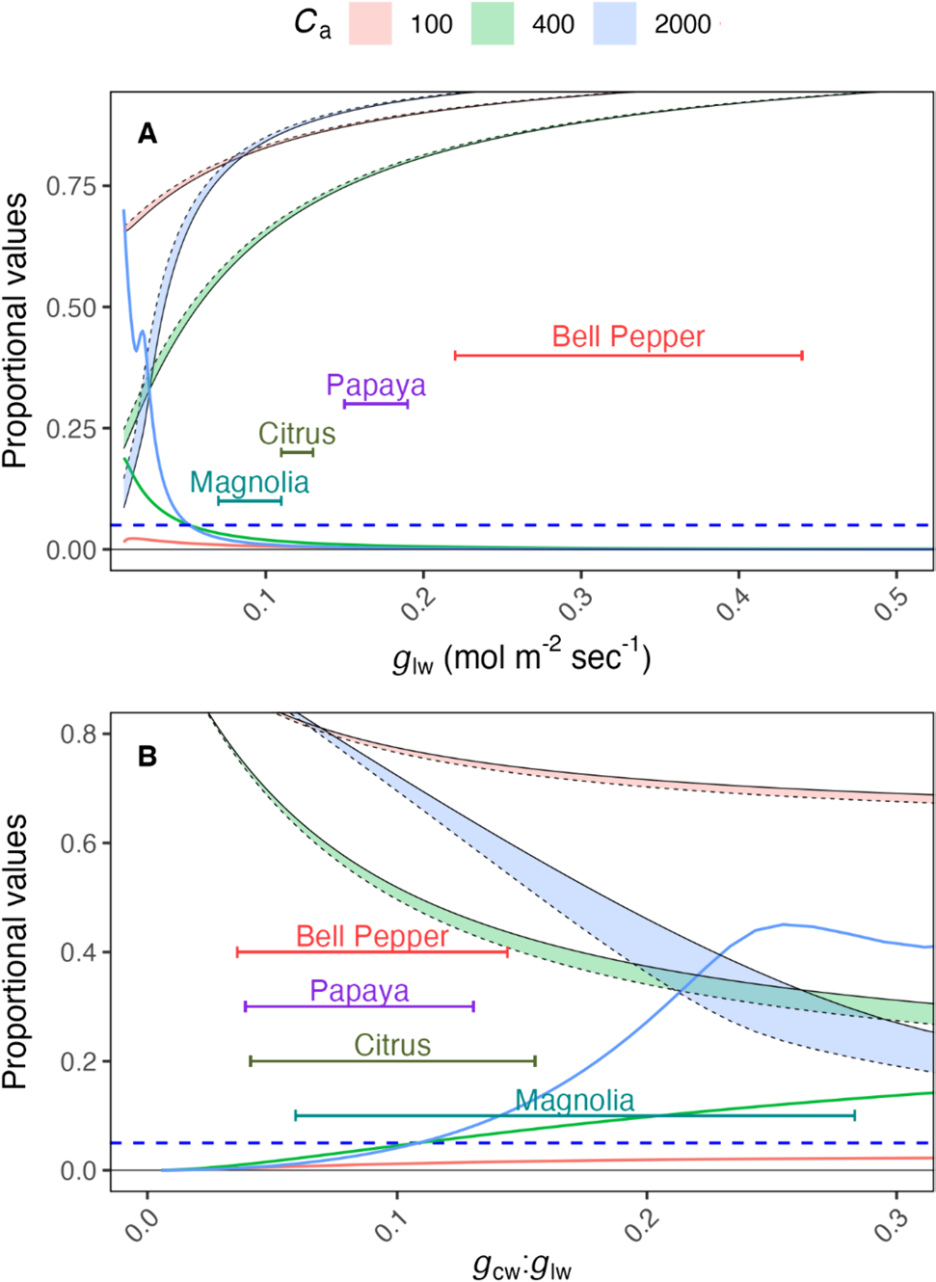
Miscalculation in estimates of leaf internal CO_2_ (*C*_i_) concentrations over a range of low leaf conductance to water (*g*_lw_) values considering the impact of cuticular conductance to water (*g*_cw_) in the estimation of *g*_sw_ and the resulting *g*_tc_ and *C*_i_. Proportional *C*_i_ values are proportional to the value when *g*_sw_ = 1 mol m^−2^ s^−1^. A shows the impact of accounting for *g*_cw_ on the estimate of *C*_i_, expressed as a proportion of the *C*_i_ estimate at *g*_lw_ = 1.0 mol H_2_0 m^−2^ s^−1^. B shows the same values as a proportion of *g*_cw_ to *g*_lw_. The light red, green, or blue ribbons show the gap between the corrected and uncorrected *C*_i_ values, bounded by the uncorrected *C*_i_ estimate (solid black line) and the corrected estimate (dashed black line) proportional to the corrected value at the same* g*_sw_. The solid lines corresponding to the bands show the proportional misestimation of *C*_i_ due to not accounting for *g*_cw_. The horizontal error bars show ± 1 standard deviation of maximum *g*_cw_:*g*_lw_ in a series of five steady-state *A*/*C*_i_ curves for each species. Assumptions, in this case, are *V*_cmax_ = 50 µmol m^−2^ s^−1^, *C*_a_ = 400 µmol mol^−1^, and VPD = 1.5 kPa

We modeled the impact of accounting for *g*_cw_ for a more accurate estimate of *g*_tc_ (Fig. 2), using 0.0056 mol m^−2^ s^−1^ estimate of *g*_cw_ from papaya as the case, because it fell toward the middle of the range of the four species assessed in this study (See below). As *g*_lw_ declined, the proportional misestimation of *C*_i_ increased exponentially (Fig. 1), this can be expressed as a proportion of *g*_cw_:* g*_lw_, resulting in a much greater alignment of the species tested and that the slope the response of misestimation due to the assumption that *g*_sw_ = *g*_lw_ is much slower than in response to *g*_lw_ alone. These results suggest that including *g*_cw_ in the calculation of *C*_i_ will be important in any context where *g*_cw_ is greater than 10% of *g*_lw_. Additionally, even species with relatively high *g*_lw_ (eg. *C. annuum* and *C. papaya*) reached sufficiently high *g*_cw_:*g*_lw_ to cause very large (> 50%) misestimations of *C*_i_. The overall response of misestimation of *C*_i_ can be seen in Supplemental Fig. 1, where increasing *g*_cw_:*g*_lw_ increases the misestimation of *C*_i_ with moderate impacts from *A*, *E*, and *C*_a_.

### ***Noise in the measurement of leaf conductance to water impacts estimation of internal CO***_***2***_*** at low conductance***

**Fig. 3:**
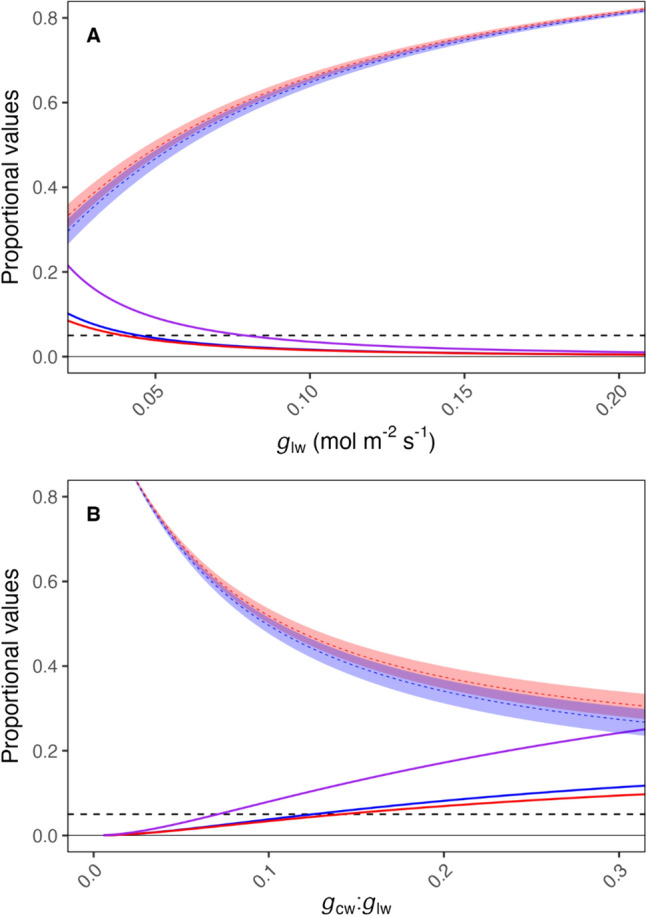
Uncertainty due to instrumental noise and miscalculation in estimates of leaf internal CO_2_ (*C*_i_) concentrations over a range of low leaf conductance to water (*g*_lw_) values considering the impact of cuticular conductance to water (*g*_cw_) and technical uncertainty in the estimation of *g*_lw_. A shows *C*_i_ estimate and potential proportional miscalculation in response to *g*_lw_. B shows the same variables in response to the ratio of *g*_cw_ to *g*_lw_. *C*_i_ values are expressed as a proportion of the *C*_i_ estimate at *g*_lw_ = 1.0 mol H_2_O m^−2^ s^−1^. Proportional error values are the difference between the uncorrected and the corrected values divided by the corrected value. Red indicates uncorrected estimates, and blue indicates corrected estimates. Dashed lines represent the estimate, while the ribbons indicate the range ± std. dev. Solid lines represent the proportional error in the *C*_i_ estimate. The purple line represents the maximum possible combined error from uncertainty in measuring *g*_lw_ and not accounting for *g*_cw_. This scenario considers *V*_cmax_ = 50 µmol m^−2^ s^−1^, *C*_a_ = 400 µmol mol^−1^, and VPD = 1.5 kPa. ambient CO_2_, *g*_cw_ = 0.0056 µmol m^−2^ s^−1^, and the standard deviation of *g*_lw_ = 0.00415 mol m^−2^ s^−1^. The error in the *g*_lw_ is based on empty chamber measurements using an LI-6800 infrared gas analyzer (LI-COR Biosciences, Lincoln, NE). The dashed horizontal line denotes a proportion of 0.05 (5% miscalculation or uncertainty)

Noise is the random unexplained error associated with any measurement. Any instrument will have some noise associated with the estimation of any variable. We estimated the impact of noise on the IRGA instrument’s estimation of *g*_lw_ by summarizing the *g*_lw_ estimates across several empty-chamber rapid *A*/*C*_i_ curves, which had been gathered to calibrate the leaf-sample curves. Because an empty, sealed chamber with no sample is known to have a conductance of 0, we could then record the conductance to estimate the precision of the estimate of 0. The standard deviation of *g*_lw_ was 0.00415 mol m^−2^ s^−1^. Given that the true value of *g*_lw_ may easily be ± one standard deviation of the estimate at any given point, we added and subtracted this instrumental noise value from the g_lw_ estimate to provide a range of possible “true values” and re-calculated *A* and *C*_i_. Instrumental noise in measuring *g*_lw_ greatly impacts estimates of *C*_i_ at low *g*_lw_ values and compounds the possible misestimation of *C*_i_ when not accounting for *g*_cw_ (Fig. 3).

### ***Considering cuticular conductance to water and instrumental noise alters A/C***_i_*** response estimates***

**Fig. 4:**
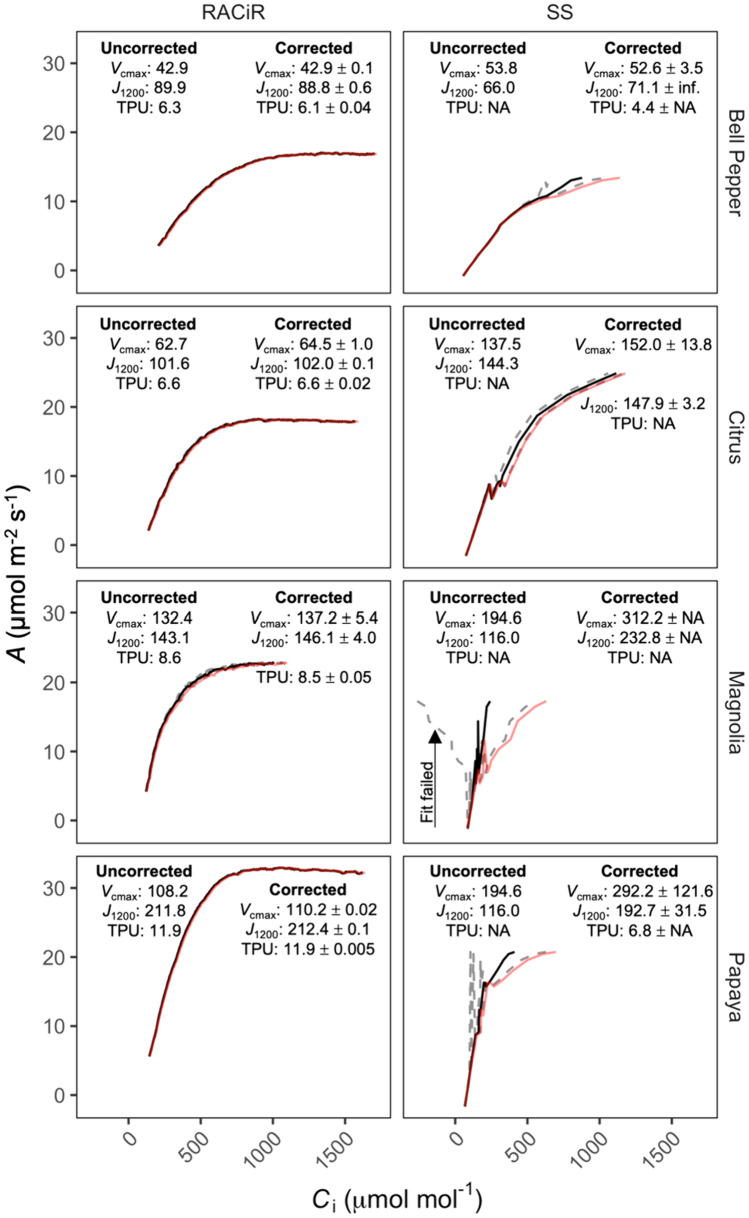
Steady-state (SS) and rapid *A*/*C*_i_ (RACiR) curves for bell pepper (*Capsicum annuum*), citrus (*Citrus* x *sinensis*), magnolia (*Magnolia grandiflora*), and papaya (*Carica papaya*). Each panel shows a single *A*/*C*_i_ curve. The curves with the lowest leaf conductance to water (*g*_lw_) for each method for each species were selected. Red lines represent an uncorrected curve in which *g*_lw_ is assumed to be equivalent to stomatal conductance to water. Solid black lines show the same curve after correcting *C*_i_ estimates accounting for the species-specific cuticular conductance. Dashed lines represent the range after accounting for instrumental noise in the measurement of stomatal conductance. The standard deviation of *g*_lw_ = 0.00415 mol m^−2^ s^−1^, based on empty chamber measurements in an LI-6800 infrared gas analyzer (LI-COR Biosciences, Inc., Lincoln, NE, USA)

We measured the *g*_cw_ of magnolia, papaya, citrus, and bell pepper, and found a wide range among these species from 0.0045 to 0.0076 mol m^−2^ s^−1^ (Table 2). Although the *g*_cw_ estimates were within the range of the standard deviation of any single measurement of *g*_lw_, the standard deviation of the estimates of *g*_cw_ were between 0.0009 and 0.0024. The greater precision of the estimate of *g*_cw_ results from the approach to measuring *g*_cw_ which uses the average of *g*_lw_ over 5 min with measurements every 7 s Thus, each *g*_cw_ measurement resulted from the mean of 42 *g*_lw_ measurements. We considered the *g*_cw_ of each species, as well as possible instrumental noise, and applied these to the RACiR and SS *A/C*_i_ curves with the lowest observed conductance for each species (Fig. 4). The impact of accounting for *g*_cw_ and instrumental noise in the estimation of *g*_lw_ varied by species and by the *A*/*C*_i_ curve method, suggesting that in many cases constant-ramping (RACiR) curves are more precise than SS curves, and that under some conditions SS curves produced estimates within 1 standard deviation of *g*_lw_ of unrealistic *A*/*C*_i_ results.

**Table 2:** Mean leaf conductance to water (*g*_lw_) and cuticular conductance to water (*g*_cw_) of magnolia (*Magnolia grandiflora*), citrus (*Citrus sinensis*), bell pepper (*Capsicum annuum*), papaya (*Carica papaya*)

Species	*g*_lw_ (mol m^−2^ s^−1^)	*g*_cw_ (mol m^−2^ s^−1^)
Magnolia (*Magnolia grandiflora*)	0.09 ± 0.02	0.0053 ± 0.0019
Citrus (*Citrus sinensis*)	0.12 ± 0.01	0.0045 ± 0.00085
Bell pepper (*Capsicum annuum*)	0.33 ± 0.11	0.0076 ± 0.0015
Papaya (*Carica papaya*)	0.17 ± 0.02	0.0056 ± 0.0024

*g*_lw_ values are the mean and standard deviation of measurements of five leaves during a steady-state *A*/*C*_i_ curve. *g*_cw_ are the mean and standard deviation of five leaves, estimated according to the method of Márquez et al. (2022)

### ***RACiR versus steady-state A/C***_***i***_*** curves***

Overall, results from SS and RACiR curves were similar (Table 3). Notable differences are the smaller *R*_dark_ estimates by SS than by RACiR and the small number of TPU estimates produced by RACiR, the latter was not surprising considering the lower pre-set range of *C*_a_ values for RACiR, which prompted us to gather a new data set to test whether consistent estimation of TPU was possible with RACiR (see below). In addition, for magnolia, *V*_cmax_ fitted to RACiR curves was lower than SS curves. In citrus, the value of *g*_sw_ was higher in RACiR than in SS, illustrating that stomatal attenuation in citrus is lesser when using constant ramping than SS methods, because there is less time for stomatal responses to high *C*_a_.

**Table 3:** Comparison of photosynthetic parameters as measured by steady-state (SS) and rapid *A*/C_i_ response (RACiR) curves of magnolia (*Magnolia grandiflora*), citrus (*Citrus sinensis*), bell pepper (*Capsicum annuum*), papaya (*Carica papaya*) leaves with uncorrected *g*_sw_ or corrected* g*_sw_, representing the mean value for all measurements of each curve

Photosynthetic traits	Plant species	With Uncorrected *g*_sw_		With Corrected* g*_sw_		Δ CV^3^
		RACiR	SS	RACiR	SS	
*V*_cmax_ (µmol m^−2^ s^−1^)	Magnolia	130.38 ± 10.47*^Ω^	176.51 ± 6.85	145.64 ± 7.43	220.00 ± 31.14	0.64
	Citrus	110.98 ± 6.36^Ω^	107.13 ± 5.31^Ω^	115.11 ± 6.79	111.67 ± 5.90	−0.12
	Bell Pepper	79.28 ± 9.32	69.10 ± 7.22^Ω^	79.30 ± 9.28	70.18 ± 7.42	−0.11
	Papaya	239.05 ± 18.40	244.12 ± 21.53^Ω^	258.29 ± 23.88	259.08 ± 21.61	−0.11
*J*_1200_ (µmol m^−2^ s^−1^)	Magnolia	139.97 ± 23.57^Ω^	162.44 ± 17.54	152.43 ± 21.42	200.46 ± 11.68	−1.41
	Citrus	128.08 ± 6.07^Ω^	118.00 ± 4.74	130.25 ± 6.23	120.68 ± 4.73	−0.22
	Bell Pepper	117.75 ± 10.13	104.01 ± 16.84	116.26 ± 10.44	104.89 ± 16.61	0.43
	Papaya	370.17 ± 62.48	304.38 ± 59.05	414.92 ± 67.60^1^	324.89 ± 61.27	0.14
*R*_dark_ (µmol m^−2^ s^−1^)	Magnolia	1.83 ± 0.09	1.27 ± 0.33	2.27 ± 0.19	1.78 ± 0.61	0.76
	Citrus	1.74 ± 0.13*^Ω^	0.13 ± 0.09	1.80 ± 0.13*	0.11 ± 0.10	0.92
	Bell Pepper	1.92 ± 0.18*	0.97 ± 0.26^Ω^	1.79 ± 0.17*	0.94 ± 0.26	0.66
	Papaya	2.68 ± 0.45*	0.10 ± 0.22	2.91 ± 0.44*	0.06 ± 0.25	0.96
TPU (µmol m^−2^ s^−1^)	Magnolia	NA	10.52 ± 0.26^2^	NA	10.55 ± 0.24^2^	NA
	Citrus	6.09 ± 0.12^2^	6.45 ± 0.29^2^	6.29 ± 0.11^2^	6.40 ± 0.29^2^	0.61
	Bell Pepper	5.94 ± 0.32^2^	8.23 ± 0.09^2^	5.77 ± 0.22^2^	8.19 ± 0.07^2^	−3.46
	Papaya	NA	9.75 ± 2.25^2^	NA	11.84 ± 2.26^2^	NA
*C*_itrans1_ (µmol mol^−1^)	Magnolia	338.68 ± 47.94^Ω^	302.55 ± 60.47	320.57 ± 46.87	285.05 ± 49.93	0.17
	Citrus	470.63 ± 12.51^Ω^	459.79 ± 19.66^Ω^	453.44 ± 12.91	443.98 ± 17.36	0.27
	Bell Pepper	605.33 ± 12.88^Ω^	578.92 ± 65.65	590.93 ± 13.20	570.88 ± 57.83	0.78
	Papaya	376.63 ± 32.64^Ω^	327.40 ± 71.11	355.02 ± 32.95	304.75 ± 55.31	0.49
*C*_itrans2_ (µmol mol^−1^)	Magnolia	NA	943.67 ± 89.68^2^	NA	891.66 ± 126.46^2^	NA
	Citrus	543.86 ± 27.84^2^	718.68 ± 38.78^2Ω^	510.91 ± 20.64^2^	685.32 ± 38.13^2^	0.27
	Bell Pepper	638.67 ± 7.07^2^	1229.17 ± 60.08^2^	634.96 ± 5.90*^2^	1152.88 ± 25.89^2^	0.59
	Papaya	NA	748.20 ± 161.16^2^	NA	735.05 ± 156.81^2^	NA
*g*_sw_ (mol m^−2^ s^−1^)	Magnolia	0.112 ± 0.022^Ω^	0.091 ± 0.015^Ω^	0.103 ± 0.023	0.086 ± 0.015	−0.28
	Citrus	0.153 ± 0.013*	0.120 ± 0.008^Ω^	0.149 ± 0.014*	0.116 ± 0.008	−0.36
	Bell Pepper	0.261 ± 0.023	0.328 ± 0.108^Ω^	0.256 ± 0.025	0.320 ± 0.108	0.71
	Papaya	0.217 ± 0.037	0.175 ± 0.028^Ω^	0.217 ± 0.036	0.169 ± 0.029	NA

Values are mean ± standard error. *V*_cmax_ is the maximum rate of Rubisco carboxylase activity,* J*_1200_ is the maximum rate of photosynthetic electron transport under saturating light (photosynthetic photon flux density of 1200 µmol m^−2^ s^−1^),* R*_dark_ is the daytime respiration. TPU is a triose phosphate utilization limitation rate. *C*_i_ is the intercellular concentration of CO_2_. *C*_itrans1_ represents *C*_i_ at which the transition between CO_2_ and RuBP limitations (i.e. *V*_cmax_ and *J*_1200_) occurs. *C*_itrans2_ is *C*_i_ at which the transition from RuBP to TPU limitations (i.e. *J*_1200_ and TPU) occurs. *g*_*sw*_ represents the stomatal conductance to water vapor. Except where noted n = 5

*Indicates a significant difference (*P* < 0.05) between RACiR and SS *A*/C_i_ derived parameters within *g*_sw_ corrected or uncorrected categories

^Ω^ Indicates a significant difference (*P* < 0.05) between derived parameters of *g*_sw_ corrected and uncorrected curves within RACiR or SS *A*/C_i_ curve categories

^1^Mean excludes the *J*_1200_ estimate of 1,000,000 µmol m^−2^ s^−1^, as this is considered to be an error

^2^At least one replication has missing data, which resulted when the curve fitting did not produce an estimate for that parameter

^3^Δ CV is the fold change in the coefficient of variation (standard deviation/mean) due to using RACiR versus using the steady state technique (eg. positive Δ CV means RACiR reduces CV relative to SS)

### ***Correcting for g***_***cw***_*** impacts the estimation of photosynthetic parameters***

To illustrate the effect of the *g*_cw_ on *A/C*_i_-derived parameters, both the RACiR and SS *A/C*_*i*_ curves were recomputed with corrected *g*_sw_. We then estimated the photosynthetic parameters from the recalculated *A/C*_i_ data (Table 3). For magnolia and citrus, correcting for *g*_cw_ affected *V*_cmax_ and *J*_1200_ estimates. In the case of SS curves, correcting for *g*_cw_ affected *V*_cmax_ estimates in all species except for magnolia. With corrected *g*_*sw*_, no difference was found for *R*_dark_ regardless of *A/C*_*i*_ curve type, except for citrus, which had a greater *R*_dark_ value in RACiR with corrected *g*_sw_. Interestingly, correcting *g*_sw_ decreased RACiR-derived *C*_itrans1_ for all species. However, for SS, only citrus showed a difference between corrected and uncorrected *g*_sw_*-*derived *C*_itrans1_ and *C*_itrans2_. The *g*_cw_ correction strongly decreased *g*_sw_ estimate in SS curves. However, for RACiR, only *g*_sw_ estimates in magnolia were affected by *g*_cw_ correction.

### Stomatal limitations to gas exchange

**Fig. 5:**
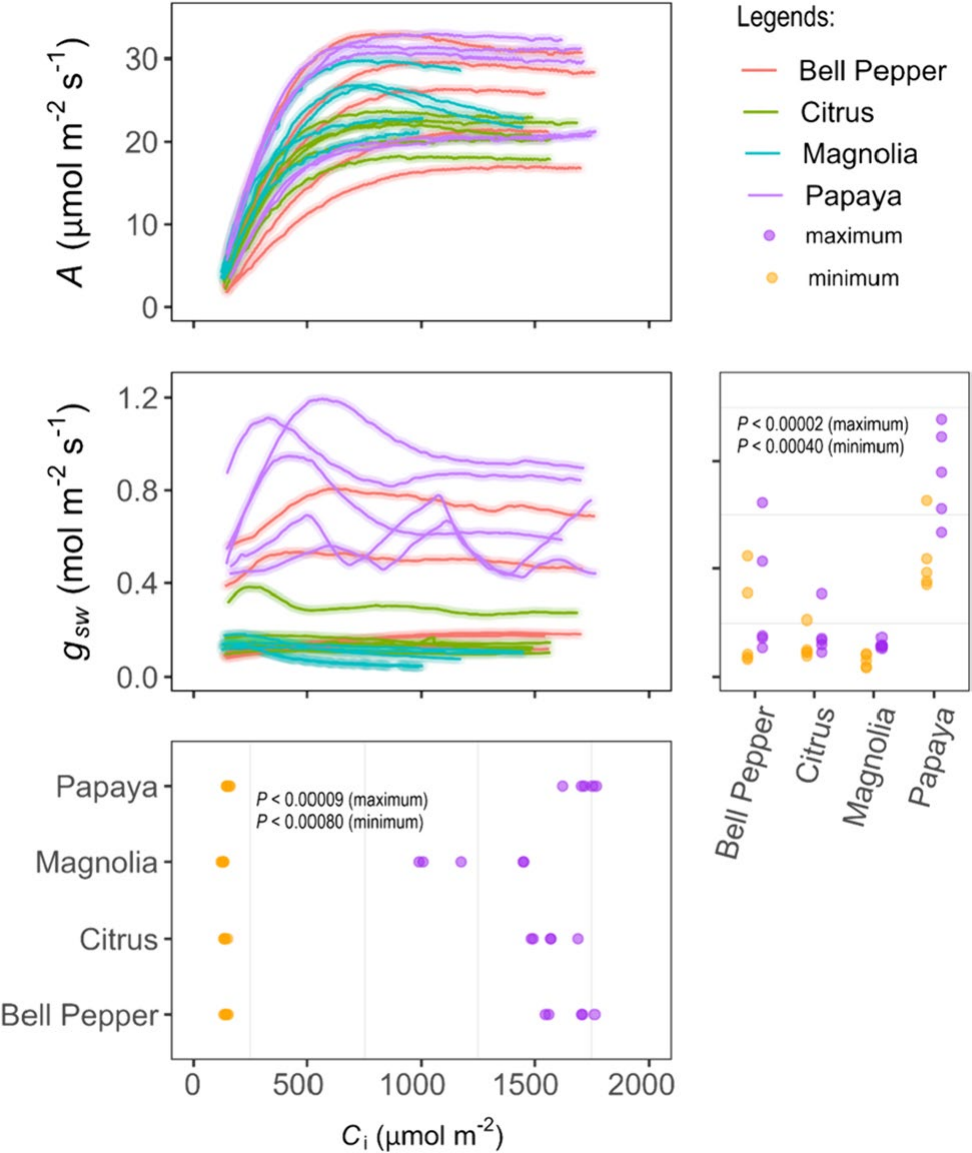
Plots of net CO_2_ assimilation (*A*) and stomatal conductance (*g*_sw_) by intercellular CO_2_ concentration (*C*_i_), and distributions of maximum and minimum *g*_sw_ and *C*_i_ of leaves of magnolia (*Magnolia grandiflora*), citrus (*Citrus sinensis*), bell pepper (*Capsicum annuum*), papaya (*Carica papaya*) using RACiR method. *P-*values indicate the results of Tukey’s HSD test

Maximum and minimum *g*_sw_ and *C*_i_ differed among species (Fig. 5). Papaya had the greatest *g*_sw_ followed by bell pepper, citrus, and magnolia. The same trend was found for *C*_i_. Each species’ *g*_sw_ responded differently to changing *C*_a_ in the RACiR curves, with some exhibiting reductions in *A* due to low *g*_sw_. In magnolia, two curves decreased *A* noticeably as *C*_a_ exceeded 700 µmol mol^−1^ (Fig. 5). Moreover, in all the RACiR curves in magnolia, *g*_sw_ decreased or remained low over time, regardless of *C*_i_ concentration. In bell pepper, *g*_sw_ increased from *C*_a_ of 380 µmol mol^−1^ to 475 µmol mol^−1^_,_ while higher *C*_a_ values decreased *g*_sw_. In citrus, *g*_sw_ decreased as *C*_a_ increased above ~ 400 µmol mol^−1^.

### ***RACiR curves with wide C***_***a***_*** range***

To test the range of *C*_i_ attainable using constant-ramping, we performed separate curves with elevated *C*_a_ up to 2000 µmol mol^−1^ (Table 4). Overall, the *g*_sw_ pattern among species was the same as for previous measurements. Moreover, TPU fitted by RACiR curves was observed for all species except for one curve in magnolia (Table 4 and Fig. 4). Correcting for *g*_cw_ increased the *V*_cmax_ estimate in all species, except citrus. *J*_1200_ was only impacted in magnolia. *R*_dark_, TPU, and *C*_itrans2_ were decreased in bell pepper and papaya while *C*_itrans1_ and *g*_sw_ were decreased in all species by correcting for *g*_cw_.

**Table 4:** Comparison of photosynthetic parameters as measured by rapid *A*/C_i_ response (RACiR) curves of magnolia (*Magnolia grandiflora*), citrus (*Citrus sinensis*), bell pepper (*Capsicum annuum*), papaya (*Carica papaya*) leaves with uncorrected and corrected *g*_sw_

Photosynthetic parameters	Plant species	Uncorrected RACiR	Corrected RACiR
*V*_cmax_ (µmol m^−2^ s^−1^)	Magnolia	98.43 ± 9.78*	102.16 ± 10.12
	Citrus	84.36 ± 5.89	85.13 ± 6.40
	Bell pepper	72.65 ± 11.13*	73.74 ± 11.41
	Papaya	94.42 ± 10.40*	96.14 ± 10.80
*J*_1200_ (µmol m^−2^ s^−1^)	Magnolia	153.85 ± 12.93*	156.11 ± 13.03
	Citrus	130.95 ± 8.02	127.57 ± 8.24
	Bell pepper	147.43 ± 20.08	146.84 ± 20.26
	Papaya	170.01 ± 21.23	170.54 ± 21.40
*R*_dark_ (µmol m^−2^ s^−1^)	Magnolia	2.35 ± 0.38	2.31 ± 0.38
	Citrus	2.23 ± 0.28	2.20 ± 0.30
	Bell pepper	2.32 ± 0.22*	2.13 ± 0.23
	Papaya	2.76 ± 0.20*	2.71 ± 0.20
TPU (µmol m^−2^ s^−1^)	Magnolia	9.32 ± 0.46	8.95 ± 0.54
	Citrus	7.93 ± 0.37	7.76 ± 0.38
	Bell pepper	9.13 ± 0.88*	9.07 ± 0.89
	Papaya	9.92 ± 0.86*	9.91 ± 0.86
*C*_itrans1_ (µmol mol^−1^)	Magnolia	419.65 ± 59.03*	399.57 ± 55.81
	Citrus	429.89 ± 16.93*	408.48 ± 18.86
	Bell pepper	647.60 ± 51.68*	623.45 ± 50.42
	Papaya	465.60 ± 21.73*	452.00 ± 21.62
*C*_itrans2_ (µmol mol^−1^)	Magnolia	656.26 ± 36.50	683.98 ± 70.26
	Citrus	756.38 ± 44.56	759.47 ± 33.02
	Bell pepper	1052.75 ± 124.40*	1035.95 ± 123.58
	Papaya	866.76 ± 106.65*	849.38 ± 105.04
*g*_sw_ (mol m^−2^ s^−1^)	Magnolia	0.113 ± 0.011*	0.108 ± 0.011
	Citrus	0.174 ± 0.034*	0.170 ± 0.034
	Bell pepper	0.340 ± 0.120*	0.332 ± 0.120
	Papaya	0.742 ± 0.090*	0.736 ± 0.090

Values are mean ± standard error. *V*_cmax_ is the maximum rate of Rubisco carboxylase activity,* J*_1200_ is the maximum rate of photosynthetic electron transport under saturating light (photosynthetic photon flux density of 1200 µmol m^−2^ s^−1^),* R*_d_ is the daytime respiration.* C*_i_ is the intercellular concentration of CO_2_. *C*_itrans1_ represents C_i_ at which the transition between CO_2_ and RuBP limitations (i.e. *V*_cmax_ and *J*_1200_) occurs. *C*_itrans2_ is C_i_ at which the transition from RuBP to TPU limitations (i.e. *J*_1200_ and TPU) occurs. *g*_*sw*_ represents the stomatal conductance to water vapor

## Discussion

### ***Species-specific leaf conductance characteristics and their implications for A/C***_***i***_*** curves***

Leaf conductance and stomatal behavior are known to vary across environmental conditions and species (Jones et al. 2009; Cabrerizo and Marañón 2022). Through the characterization of species-specific leaf conductance, researchers can gain valuable insights into the functional traits and adaptations that contribute to the successful adaptation of plants in diverse environments (Mott and Buckley 2000; Franks et al. 2009). For this study, we selected four plant species with distinct leaf conductance characteristics: bell pepper and papaya, exhibiting high *g*_lw_, citrus with intermediate *g*_lw_, and magnolia with the lowest *g*_lw_. *g*_cw_ values were 1–2 orders of magnitude less than the *g*_lw_ (Table 2). *g*_cw_:*g*_lw_ helped make species more comparable in terms of misestimation of *C*_i_, and a ratio of only 0.1 was required to exceed 5% misestimation of *C*_i_ whenever *C*_a_ was 400 ppm or greater. This ratio could be used to assess the risk of large *C*_i_ misestimation, because the risk of misestimation could not be assessed using *g*_lw_ alone. The risk of misestimation of *C*_i_ increased with *C*_a_, however, the 5% misestimation point is exceeded at approximately the same *g*_lw_ for *C*_a_ of both 400 and 2,000 ppm. This misestimation has implications for the interpretation of *A*/*C*_i_ curves, as demonstrated by the problematic nature of some of the uncorrected SS curves (Fig. 4). Our findings emphasize the necessity of accounting for *g*_cw_ in accurately estimating *C*_i_ and highlight the potential impact of misestimation on the interpretation of* A*/*C*_i_ responses.

### ***Impact of accounting for g***_***cw***_*** on A/C***_***i***_*** fitted parameters***

The cuticle has been known to impact the estimation of photosynthetic parameters by impacting estimates of *C*_i_ (Hanson et al. 2016). According to previous reports, *g*_cw_ influences the calculation of *C*_i_ (Boyer 2015a; Tominaga et al. 2018). In the present study, recalculated *A/C*_i_ curves (both SS and RACiR) using the *g*_cw_ values obtained from the red-light method (Márquez et al. 2021) indicate that considering *g*_cw_ does modify *C*_i_ estimates. The extent of the effect varies among species and is more pronounced when the *g*_cw_:*g*_lw_ ratio is high or the *g*_sw_ is low, which results in a decrease in *C*_i_. Lamour et al. (2022) proposed that the ratio of *g*_cw_:*g*_lw_ could be important in predicting the misestimation of *C*_i_. The present results support and extend that hypothesis by demonstrating that even high conductance species may have sufficiently high *g*_cw_:*g*_lw_ ratios to have strong impacts on the misestimations of *C*_i_. Papaya was the least affected on average, as it exhibited the highest *g*_sw_ during measurements, whereas magnolia, with low *g*_sw_, showed significant changes in most photosynthetic parameters. These findings underscore the importance of *g*_cw_ in estimating photosynthetic traits, as ignoring *g*_cw_ can introduce a bias that incorrectly increases estimates of *C*_i_. The data presented in Table 3 and Table 4 indicate a significant increase in *V*_cmax_, *J*_max_, *R*_dark_, and *C*_itrans1_ when the *g*_cw_:*g*_sw_ ratio is high, which could occur in any of the species assessed when *g*_sw_ was low within the range for that species. Thus, plant conditions of water deficit, supra- or suboptimal temperatures, or other adverse growing conditions are likely to induce sufficiently low *g*_sw_ as to necessitate the estimation of *g*_cw_ to estimate accurate photosynthetic parameters.

### Constant-ramping A/Ci method improves parameter resolution and avoids some stomatal limitations

In previous studies, comparisons between the RACiR and SS methods have indicated that the *A*/*C*_i_ fitted parameters, *V*_cmax_ and *J*_max_, show no significant differences in a range of species (Stinziano et al. 2017, 2019b; Coursolle et al. 2019; Lawrence et al. 2019; Saathoff and Welles 2021; Lin et al. 2023). In our study, the results of *A*/*C*_i_ curves obtained using both the SS and RACiR methods were similar, with more precise estimates of *C*_itrans1_ observed with the RACiR method, as seen in the smaller standard deviation in *C*_itrans1_ in RACiR than SS for all species, with and without correction of *C*_i_ (Table 3). For example, the reductions in standard deviation represent a 16% decrease in the coefficient of variation (standard deviation/mean) for magnolia (the smallest change) and a 78% decrease in the coefficient of variation for bell pepper (the largest change). However, a notable difference was found in the *g*_sw_ values, where the SS curves showed a greater decrease in *g*_sw_ compared to the RACiR curves (Fig. 5). The likely reason for these differences is that the RACiR curves allowed less time for *g*_sw_ to decrease before reaching the critical threshold. SS curves are measured slowly relative to the time scale of leaf CO_2_ attenuation of *g*_sw_ (Merilo et al. 2015). This leads to the reduction of *g*_sw_ in some curves below that required for reliable estimates of *C*_i_. We do note, however, that the traditional approach to *A*/*C*_i_ curves—starting near atmospheric CO_2_, going down, then up such as in Busch (2018) may not be the best option for maximizing *g*_sw_ during the SS. Starting at low CO_2_ and monotonically increasing CO_2_ may result in better *A*/*C*_i_ estimates (Sharkey 2019), though our initial attempts were not successful with this approach in terms of avoiding the *g*_sw_ crash in citrus and magnolia.

To assess the *A*/*C*_i_ relationship, it is ideal to achieve a wide range of *C*_i_ values. The RACiR method achieved as wide a range of *C*_i_ as the SS method, though low conductance magnolia limited the *C*_i_ range achieved (Fig. 2). These findings align with the results reported by Lin et al. (2023), who found that measuring SS *A*/*C*_i_ and RACiR curves in low conductance species posed challenges due to their low *g*_sw_ values. Consequently, driving *C*_i_ to a very high level is difficult or even impossible in these species. In the present study, at higher *C*_a_ values *g*_*sw*_ decreased (Fig. 5). In this case, the advantage of constant ramping lies in that its short time “beats” the speed of stomatal attenuation, affording the ability to avoid stomatal impacts on the shape of the *A*/*C*_i_ curve, making TPU estimation more feasible in low conductance species.

Stomatal attenuation can particularly affect the estimation of TPU, as it may induce changes in *A* at high *C*_i_ that are limited by *g*_sw_, rather than by enzymatic limitations, despite the similarity in shape of the curve to the expected shape with TPU limitations, as is seen in Fig. 4 in papaya and bell pepper. Plants are not typically TPU-limited under ambient conditions (Sage and Sharkey 1987; Sharkey 2019; Ellsworth et al. 2015), and TPU limitation is most easily seen by elevating the rate of photosynthesis through increased light or CO_2_ or decreased O_2_ partial pressure (Sharkey et al. 1986) such that *A* is increased by 10% or 20% relative to ambient conditions (Kirschbaum 2011). Our initial comparison was between RACiR and SS fitted photosynthetic parameters with *C*_a_ maxima of 900 and 2000 µmol mol^−1^, respectively. In this comparison, SS curves yielded a greater number of TPU estimates than RACiR curves. However, when RACiR was performed with a maximum *C*_a_ of 2000 µmol mol^−1^, TPU estimates were achieved in all species (Table 4). Thus, constant-ramping methods can consistently estimate TPU when a sufficiently high maximum *C*_a_ are used.

In addition to its increased speed, the utilization of a constant ramping technique, such as RACiR, offers the advantage of improved resolution of parameter estimates. This improvement stems from the reduction in parameter uncertainty that can be achieved by having a larger amount of data available for curve fitting (Saathoff and Welles 2021). It has been observed that the accuracy of parameter estimation is directly influenced by both the number of data points and the accuracy of the gas exchange data used. When working with small and noisy data sets, obtaining robust parameter estimates can be particularly challenging (Sharkey et al. 2007; Wang et al. 2017). In our study, the SS *A*/*C*_i_ curve data consisted of only 16 data points, while the RACiR curves comprised significantly higher numbers (270 and 600 data points at 900 and 2000 µmol mol^−1^ *C*_a_, respectively). Our findings indicate the larger data set obtained by the RACiR technique reduced parameter uncertainty in comparison to SS.

The uncertainty of instrumental noise in *g*_lw_ estimates has the potential to further compound the challenges of low conductance. Accounting for *g*_cw_ can mitigate misestimation of *C*_i_, and if *g*_cw_ is accounted for, the potential error due to instrumental noise only exceeds 5% at a *g*_cw_:* g*_lw_ ratio of 0.13. However, constant-ramping methods ameliorate the impact of instrumental noise on the estimation of photosynthetic parameters due to the substantial number of measurements collected. While individual measurements of *g*_lw_ may deviate by approximately ± 0.00415 mol m^−2^ s^−1^ from the true value, the cumulative effect of numerous measurements diminishes the impact of the error on the overall results. With an increased number of measurements, the net effect tends toward zero, rendering the influence of instrumental noise inconsequential in the approach that employs the larger dataset. This is reflected in the very small impact of the error of *g*_lw_ on parameter estimates in RACiR relative to SS curves (Fig. 3). Thus, accounting for *g*_cw_ and utilizing constant-ramping methods significantly enhance the accuracy and reliability of *A*/*C*_i_ parameter estimates.

## Conclusions

Low conductance presents a challenge to the estimation of photosynthetic parameters via gas exchange due to the misestimation of *C*_i_. This work demonstrates that not accounting for *g*_cw_ can lead to a misestimation of *C*_i_, which compounds to the estimation of parameters based on *C*_i_, leading to underestimation of *V*_cmax_ and *J*_max_. Instrumental noise can further compound errors when using a steady-state approach to *A*/*C*_i_ measurement. The ratio of *g*_cw_:*g*_lw_ is useful in assessing the risk of misestimating *C*_i_. Although this challenge was expected for low-conductance species, this work showed that high-conductance species, like *C. papaya*, can also exhibit high risk of overestimating *C*_i_. Thus, practices to improve the estimation of *C*_i_ include using constant-ramping methods and measuring and accounting for *g*_cw_ on at least a species basis. Using these approaches will improve the accuracy of *A*/*C*_i_-based photosynthetic parameter estimation, especially under conditions that reduce *g*_sw_.

### Supplementary Information

Below is the link to the electronic supplementary material.

Supplementary file1 (R 32 kb)

Supplementary file2 (R 12 kb)

Supplementary file3 (R 9 kb)

Supplementary file4 (R 6457 kb)

Supplementary file5 (DOCX 247 kb)

## Data Availability

The data that support the findings of this study are available from the corresponding author upon reasonable request.
