# Supplementary material for: Accurate photosynthetic parameter estimation at low stomatal conductance: effects of cuticular conductance and instrumental noise
Source: Photosynth Res. 2024 May 3;160(2-3):111–24. doi: 10.1007/s11120-024-01092-8 (PMC11108943; doi:10.1007/s11120-024-01092-8)
Supplement: Supplementary file 5 — Supplementary file5 (DOCX 247 kb) [file 11120_2024_1092_MOESM5_ESM.docx]

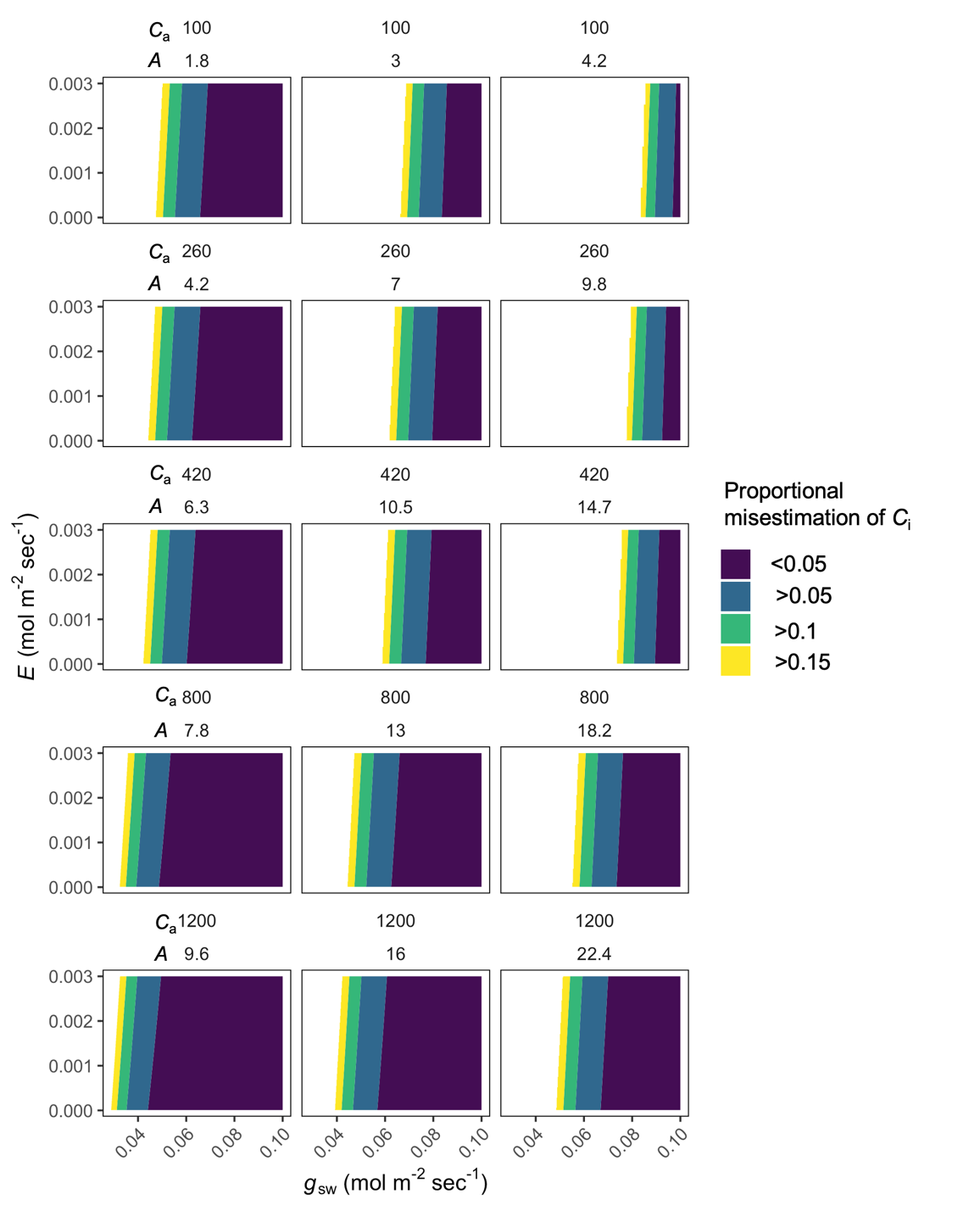


Supplemental Figure 1. Topographical heatmap of the response effect of transpiration (*E*) and stomatal conductance (*g*_sw_) under varying conditions of ambient CO_2_ concentrations (*C*_a_) and net CO_2_ assimilation (*A*). *C*_a_ concentrations were chosen to be representative of key *A*/*C*_i_ curve components. For each *C*_a_ value a characteristic *A* value was selected, and values +/- 30% of the characteristic value were also considered.
